# Mechanisms Underlying C-type Inactivation in Kv Channels: Lessons From Structures of Human Kv1.3 and Fly Shaker-IR Channels

**DOI:** 10.3389/fphar.2022.924289

**Published:** 2022-06-27

**Authors:** Seow Theng Ong, Anu Tyagi, K. George Chandy, Shashi Bhushan

**Affiliations:** ^1^ LKCMedicine-ICESing Ion Channel Platform, Lee Kong Chian School of Medicine, Nanyang Technological University, Singapore, Singapore; ^2^ School of Biological Sciences, Nanyang Technological University, Singapore, Singapore; ^3^ Singapore and Nanyang Institute of Structural Biology, Nanyang Technological University, Singapore, Singapore

**Keywords:** voltage-gated potassium (Kv) channels, C-type inactivation, slow inactivation, Kv1.3, hydrogen bond network, cryo-EM, dalazatide, Shaker-IR

## Abstract

Voltage-gated potassium (Kv) channels modulate the function of electrically-excitable and non-excitable cells by using several types of “gates” to regulate ion flow through the channels. An important gating mechanism, C-type inactivation, limits ion flow by transitioning Kv channels into a non-conducting inactivated state. Here, we highlight two recent papers, one on the human Kv1.3 channel and the second on the *Drosophila* Shaker Kv channel, that combined cryogenic electron microscopy and molecular dynamics simulation to define mechanisms underlying C-type inactivation. In both channels, the transition to the non-conducting inactivated conformation begins with the rupture of an intra-subunit hydrogen bond that fastens the selectivity filter to the pore helix. The freed filter swings outwards and gets tethered to an external residue. As a result, the extracellular end of the selectivity filter dilates and K^+^ permeation through the pore is impaired. Recovery from inactivation may entail a reversal of this process. Such a reversal, at least partially, is induced by the peptide dalazatide. Binding of dalazatide to external residues in Kv1.3 frees the filter to swing inwards. The extracellular end of the selectivity filter narrows allowing K^+^ to move in single file through the pore typical of conventional knock-on conduction. Inter-subunit hydrogen bonds that stabilize the outer pore in the dalazatide-bound structure are equivalent to those in open-conducting conformations of Kv channels. However, the intra-subunit bond that fastens the filter to the pore-helix is absent, suggesting an incomplete reversal of the process. These mechanisms define how Kv channels self-regulate the flow of K^+^ by changing the conformation of the selectivity filter.

## Introduction

Voltage-gated potassium (Kv) channels form K^+^-selective pores that span cell membranes. In humans, 40 genes encode 12 sub-families of Kv channels (Kv1-Kv12) (Alexander et al., 2019). Kv channels activate with membrane depolarization and allow the efflux of K^+^ through the open channel pore. Ion flow is then curtailed by time-dependent entry of the channels into non-conducting inactivated states (Choi et al., 1991; Hoshi and Armstrong, 2013; Armstrong and Hollingworth, 2018). This temporal regulation of K^+^ flow is essential for Kv channels to regulate electrical excitability in neurons and cardiac muscle, and modulate signaling cascades required for homeostasis and activation in non-excitable cells, (Yellen, 2002; Cahalan and Chandy, 2009; Wulff et al., 2009; Jan and January 2012; Feske et al., 2015).

**FIGURE 1:**
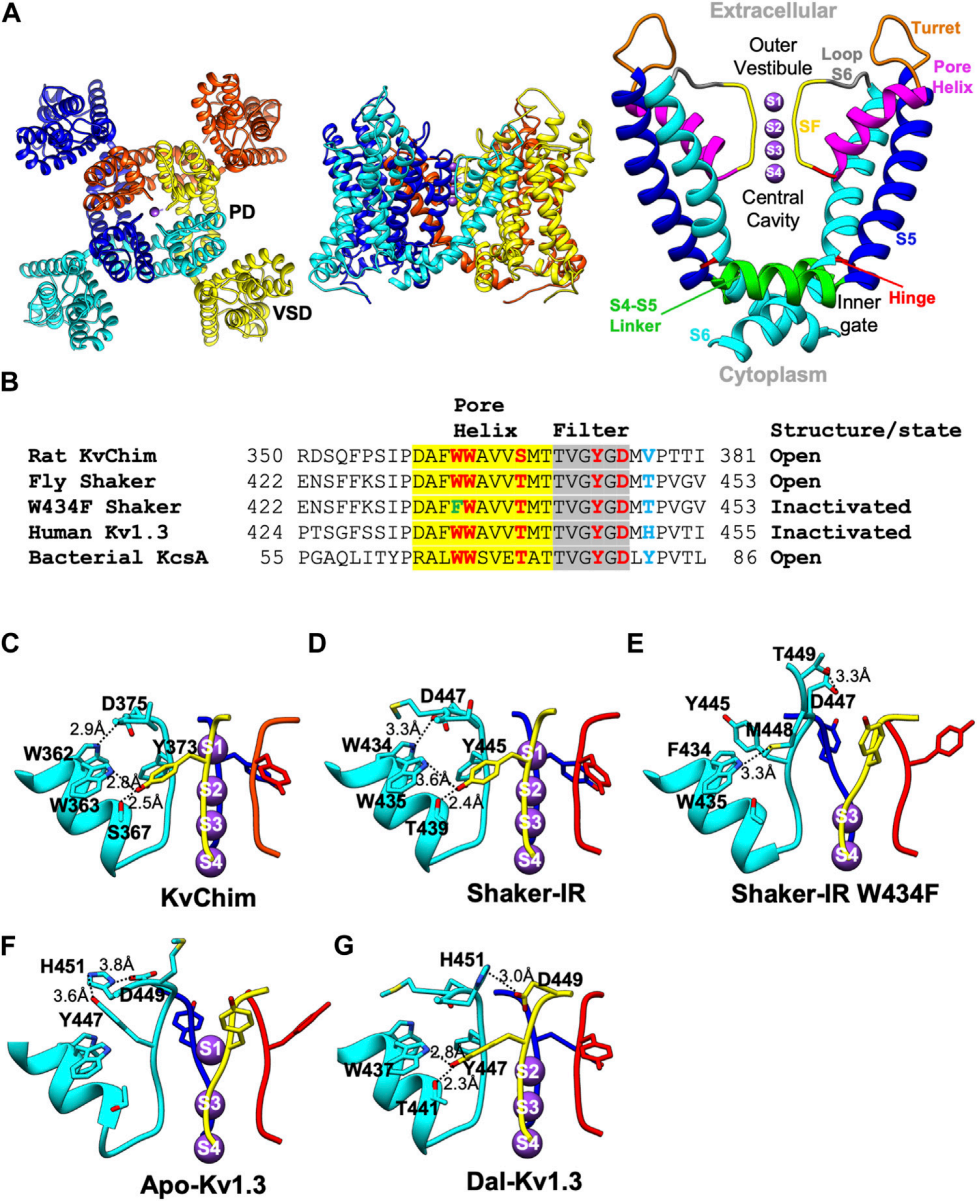
Structures of Kv channels in open-conducting, C-type open-inactivated and peptide-bound conformations. **(A)** Structure of KvChim (PDB:2R9R) viewed from the extracellular (left) and membrane planes (middle). The pore domain (PD) shown on the right is formed by S5 and S6 helices together with a P-loop consisting of a turret, pore-helix, SF and loop-to-S6. In the channel’s pore, the inner gate, central cavity, SF, outer vestibule, hinge and S4-S5 linker are shown. **(B)** Amino acid sequence alignment of P-loops of KvChim, Shaker-IR, Shaker-IRW434F, human Kv1.3 and bacterial KcsA. Residues involved in hydrogen bond networks are highlighted. **(C–G)** Hydrogen-bond networks in KvChim, PDB:2R9R; Shaker-IR, PDB:7SIP; Shaker-IR W434F, PDB:7SJ1; Apo-Kv1.3, PDB:7WF3, and Dal-Kv1.3, PDB:7WF4. Individual subunits are colored cyan, blue, red, and yellow. Selected residues are shown for clarity. Distances between hydrogen-bonded residues are shown. K^+^ (purple spheres) are shown at K^+^-binding sites, which are numbered.

High-resolution structures determined by X-ray crystallography or cryogenic-electron microscopy (cryo-EM) have provided deep molecular insights into the closed and open conformations of Kv channels (Zhou et al., 2001; Long et al., 2005; Alabi et al., 2007; Long et al., 2007; Jensen et al., 2010; Jensen et al., 2012; Kim and Nimigean, 2016; Whicher and Mackinnon, 2016; Pau et al., 2017; Sun and Mackinnon, 2017; Wang and Mackinnon, 2017; Sun and Mackinnon, 2020; Kise et al., 2021). The description below is based on the structure of the rat Kv1.2-Kv2.1 paddle chimera (KvChim) (PDB: 2R9R) (Figures 1A,B). Figure 1B shows a sequence alignment through the P-loop of KvChim and four other channels discussed in this review. Kv channels are tetramers with each monomer containing six transmembrane helices (S1–S6) and a pore-forming loop (P-loop) (Figure 1A). In each monomer, S1–S4 helices form a voltage-sensing domain (VSD) (Figure 1A, left). A K^+^-selective pore domain (PD) situated at the center of the tetramer (Figure 1A, left) is formed by S5 and S6 helices and the P-loop consisting of a turret, pore helix, selectivity filter (SF), and pre-S6 loop (Figure 1A, right). Membrane depolarization causes positively charged residues in the S4 helix to slide outwards along the charge transfer center formed by residues in the S2 and S3 helices (Tao et al., 2010). This movement is transmitted via the S4 and S5 linker to the PD to open the inner (activation) gate (Jensen et al., 2012). The pore transitions from the closed to the open-conducting conformation. K^+^ flows into a central cavity and then through the SF into the external solution (Long et al., 2005; Long et al., 2007) (Figure 1A, right). K^+^ are hydrated in the central cavity but partially lose their hydration water when they pass through the SF. K^+^ flows through the SF in single file (knock-on conduction) by being coordinated at ion-binding sites called S1, S2, S3, and S4 (Figure 1A, right) (Jensen et al., 2010). In the open-conducting conformation of KvChim (PDB: 2R9R), a network of intra-subunit (W362-D375) and inter-subunit (W363-Y373; S367-Y373) hydrogen bonds tether the SF to the pore-helix and stabilize the outer pore (Figure 1C) (Long et al., 2007; Matthies et al., 2018).

Kv channels limit K^+^ flow through their pores by inactivating during prolonged depolarization. Inactivation can occur either rapidly or slowly (Hoshi and Armstrong, 2013). The Shaker Kv channel, which has been used as a model system to study gating mechanisms of Kv channels (Yellen, 1998, 2002; Bezanilla, 2008; Swartz, 2008; Jan and January, 2012; Tan et al., 2022), undergoes both rapid and slow inactivation (Hoshi et al., 1990; Choi et al., 1991). Rapid inactivation involves a “ball and chain” mechanism in which the N-termini of Kv subunits or accessory *β*-subunits occlude the cytoplasmic entrance to the pore (Hoshi et al., 1990; Rettig et al., 1994). Residues 6–46 in Shaker Kv channel’s N-terminus form the “ball” required for rapid (N-type) inactivation (Hoshi et al., 1990). Deletion of this region produces a channel, Shaker-IR, that inactivates solely by the slow inactivation process (Yellen et al., 1994; Ogielska et al., 1995; Liu et al., 1996; Olcese et al., 1997; Loots and Isacoff, 1998; Panyi and Deutsch, 2006, 2007; Szanto et al., 2020; Szanto et al., 2021; Tan et al., 2022). Slow inactivation, an important biophysical gating mechanism of K^+^ channels (Adelman et al., 1995; Nguyen et al., 1996; Hanson et al., 1999; Hubner and Jentsch, 2002; Yellen, 2002; Vennekamp et al., 2004; Kurata and Fedida, 2006; Leung, 2012; Faouzi et al., 2015; Pau et al., 2017), involves a change in the SF (Choi et al., 1991; Yellen et al., 1994; Liu et al., 1996; Yellen, 1998; Del Camino and Yellen, 2001; Webster et al., 2004; Panyi and Deutsch, 2006; Bezanilla, 2008; Cuello et al., 2010a; Cuello et al., 2010b; Hoshi and Armstrong, 2013; Cuello et al., 2017; Valiyaveetil, 2017; Wang and Mackinnon, 2017; Armstrong and Hollingworth, 2018; Labro et al., 2018; Li et al., 2018; Szanto et al., 2020; Li et al., 2021a; Li et al., 2021b). Slow inactivation has been proposed to occur from both closed and open states of the channel (Marom and Levitan, 1994; Olcese et al., 1997; Loots and Isacoff, 1998), and to proceed in two sequential steps involving first the closure of a gate at the external entrance to the pore (P-type inactivation) and then the stabilization of the SF gate in a closed conformation (C-type inactivation) (Loots and Isacoff, 1998). Slow inactivation likely involves a manifold of inactivated states (ensembles of shallow and deeply inactivated states), and a single slow inactivated conformation does not exist (Yang et al., 1997).

High-resolution X-ray crystallographic structures have provided a detailed picture of the conformational changes during C-type inactivation of KcsA, a bacterial pH-gated two-transmembrane K^+^ channel containing a PD but no VSD (Cuello et al., 2010a; Cuello et al., 2010b; Ostmeyer et al., 2013; Cuello et al., 2017; Labro et al., 2018; Li et al., 2018). pH-dependent opening of the inner (activation) gate in KcsA is allosterically-coupled to conformational changes in the SF underlying C-type inactivation (Cuello et al., 2010a; Cuello et al., 2010b; Labro et al., 2018). Two C-type inactivated conformations of KcsA have been described, I_1_ and I_2_ (Cuello et al., 2010a; Cuello et al., 2010b). In the I_1_ conformation, the inner gate is partially opened (Cα-Cα at T112 = 17 Å), and the rearranged backbone of G77 narrows the filter resulting in occupancy of K^+^ at sites S1, S3, and S4, with no K^+^ coordinated at site S2 (Cuello et al., 2010b). In the I_2_ conformation, the inner gate is fully opened (Cα-Cα at T112 = 23–32 Å), the outer filter narrows, and the lower filter widens by ∼1.5Å increasing the accessible area at the S3 and S4 sites (Cuello et al., 2010b). This results in loss of K^+^ coordination at the S2 and S3 sites, with K^+^ occupancy only at sites S1 and S4 (Cuello et al., 2010b). The outer vestibules of the I_1_ and I_2_ conformations are unchanged compared to the open-conducting conformation, which is different from the narrowed outer vestibule of the C-type inactivated Shaker-IR Kv channel based on electrophysiological studies (Yellen et al., 1994; Liu et al., 1996). This difference may be because the KcsA structures were obtained in the presence of an antibody, which stabilized the channel and prevented the outer vestibule from undergoing conformational changes; inactivation may have been incomplete in these structures, especially because KcsA inactivates faster in the absence of a bound antibody. This suggests that KcsA may exist in additional inactivated states beyond I_1_ and I_2_. Cross-talk between the open inner gate and the SF in KcsA is mediated by the interaction of T75 at the base of the filter with F103 in the pore-lining M2 helix (Pan et al., 2011; Labro et al., 2018). The fly Shaker Kv and human Kv1.3 channels contain threonine (T442 in Shaker, T444 in Kv1.3) and isoleucine (I470 in Shaker; I472 in Kv1.3) at positions corresponding to T75 and F103 in KcsA. Replacement of T75 with alanine abolishes KcsA’s C-type inactivation (Labro et al., 2018). in Shaker Kv, the T442A and I470L mutations slow C-type inactivation, while the I470F mutation speeds up the process (Peters et al., 2013; Labro et al., 2018). In Kv1.3, the T444A mutation does not express, but the I472A Kv1.3 mutant abolishes C-type inactivation (Zimin et al., 2010). Further, electrophysiological studies on the Shaker-IR T449A/V474C and T449A/V476C mutants show that the inner (activation) gate controls steady-state inactivation at negative membrane potentials through a series of transitions: C (closed) → O (open-conducting) → OI (open-inactivated; open inner gate and altered SF) → CI (closed-inactivated; closed inner gate and altered SF) →C (Panyi and Deutsch, 2006). These results suggest that the inner (activation) gate and the SF are coupled in KcsA, Shaker Kv and human Kv1.3 channels.

While the structural studies of KcsA have highlighted the conformational changes during pH-dependent C-type inactivation, the structural details of voltage-activated C-type inactivation in Kv channels has remained elusive. In February 2022, high-resolution structures of the human Kv1.3 channel in complex with Kvβ2, alone (Apo-Kv1.3) and bound to peptide dalazatide (Dal-Kv1.3), were published (Tyagi et al., 2022). Kv1.3 regulates membrane potential and calcium signaling in many non-excitable cells and is a validated therapeutic target for autoimmune and neuroinflammatory diseases (Cahalan and Chandy, 2009; Feske et al., 2015; Wulff et al., 2019). Kv1.3 inactivates solely through the C-type mechanism; its slow rate of recovery from inactivation causes cumulative inactivation (Cahalan et al., 1985; Busch et al., 1991; Panyi et al., 1995; Nguyen et al., 1996; Jager et al., 1998; Hanson et al., 1999; Somodi et al., 2004; Vennekamp et al., 2004; Somodi et al., 2008; Liu et al., 2021). In March 2022, high-resolution structures of Shaker-IR and the non-conducting, permanently inactivated W434F mutant were published (Tan et al., 2022). This Mini Review summarizes the cryo-EM and molecular dynamics (MD) simulation studies on human Kv1.3 (Tyagi et al., 2022) and fly Shaker-IR (Tan et al., 2022). The mechanisms outlined here could spur the characterization of disease-causing mutations that impair C-type inactivation and guide the design of drugs that target the inactivated conformation.

## Conformational Changes in the Filter Underlie C-TYPE Inactivation

**FIGURE 2:**
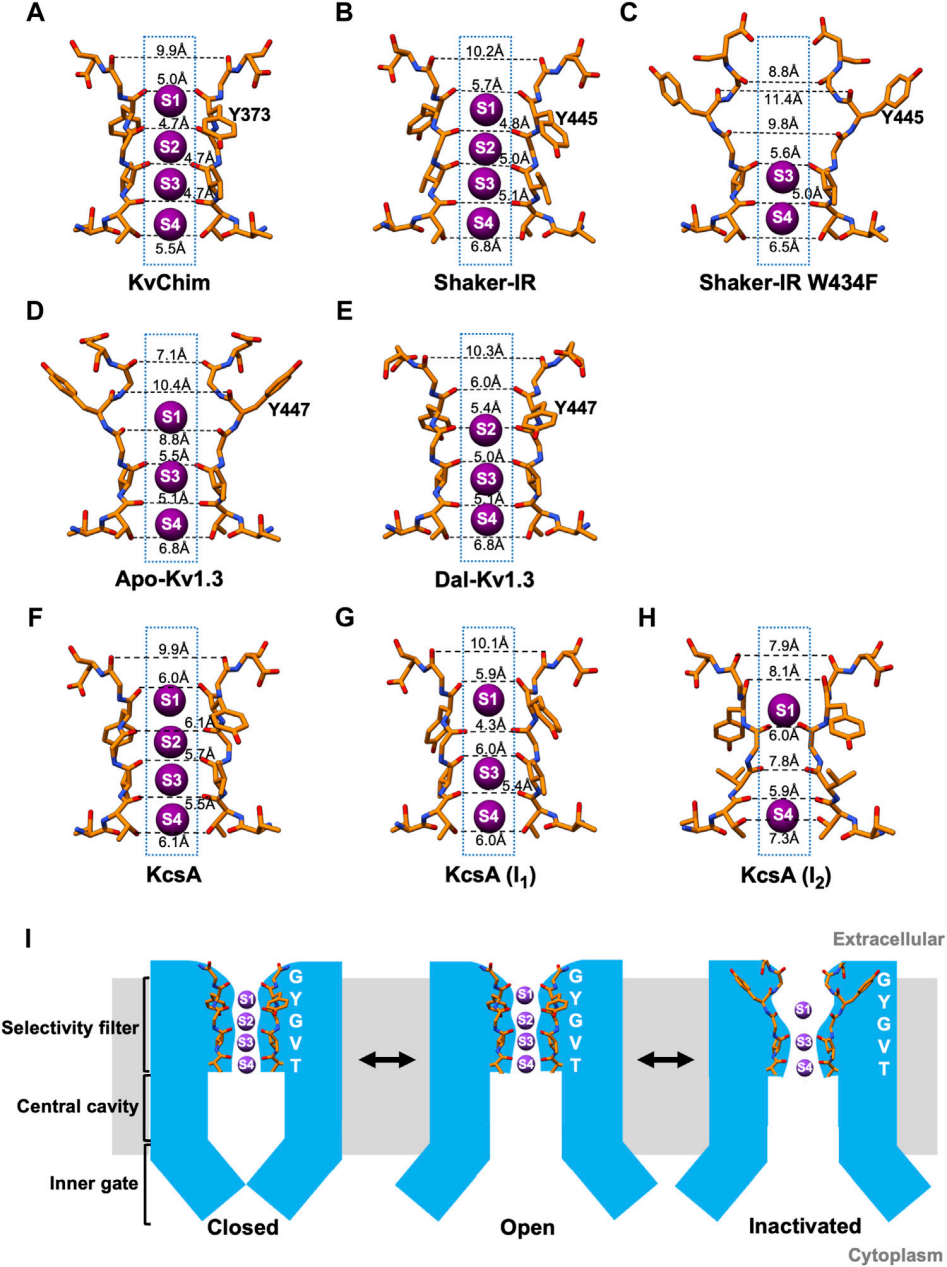
Dimensions of the selectivity filter and a schematic model of C-type inactivation. **(A–H)** Distances between carbonyl O atoms of residues in SF in KvChim, PDB:2R9R; Shaker-IR, PDB:7SIP; Shaker-IR W434F, PDB:7SJ1; Apo-Kv1.3, PDB:7WF3; Dal-Kv1.3, PDB:7WF4; KcsA open-conducting, PDB:3B5F; I_1_ open-inactivated KcsA, PDB:3F7Y; I_2_ open-inactivated KcsA, PDB:3F5W. Only two subunits are displayed for clarity. K^+^ (purple spheres) are shown at K^+^-binding sites, which are numbered. **(I)** Structure-based schematic model of closed, open and C-type inactivated states. The closed model is based on bacterial K^+^ channel KcsA (PDB:1K4C), the open-conducting model on Shaker-IR (PDB:7SIP) and Dal-Kv1.3 (PDB:7WF4), and the C-type open-inactivated model on Apo-Kv1.3 (PDB:7WF4) and Shaker-IR W434F (PDB:7SJ1).

In the wild-type Shaker-IR structure, the voltage sensors are in the depolarized position and the inner (activation) gate is open, indicating that the channel is activated (Tan et al., 2022). Earlier electrophysiological studies on Shaker channels show that the open inner gate is coupled to conformational changes in the SF associated with slow inactivation (Panyi and Deutsch, 2006; Peters et al., 2013; Labro et al., 2018; Szanto et al., 2020). Although the inner gate is open in the structure, Shaker-IR’s SF is not in a slow open-inactivated conformation, but paradoxically in the open-conducting conformation (Tan et al., 2022). K^+^ occupies all four ion-binding sites in the SF of Shaker-IR (Figure 1D), its pore dimensions (Figure 2B) are similar to KvChim (Figure 2A) and all-atom MD simulations following a voltage pulse (300 mV) show normal permeation of K^+^ through the pore (Tan et al., 2022). This result is surprising since the Shaker-IR structure is at 0 mV, which is an experimental condition in which the channel is expected to be open and C-type inactivated (OI conformation). This incongruence remains to be explained. In KcsA’s open-conducting SF conformation the inner gate is 15 Å wide; only when the inner gate widens to 17 Å does allosteric-coupling get triggered and the SF enter the I_1_ open-inactivated conformation (Cuello et al., 2010a; Cuello et al., 2010b). The dimension of Shaker-IR’s open inner gate (Cα-Cα at V474 = 15 Å; PDB: S7IP) resembles that of open-conducting KcsA (Cuello et al., 2010a; Cuello et al., 2010b). Perhaps, Shaker-IR’s inner gate has to widen further for it to enter an open-inactivated conformation. A network of intra-subunit (W434-D447) and inter-subunit (W435-Y445; T439-Y445) hydrogen bonds (Figure 1D), equivalent to those in KvChim (Figure 1C), stabilize the outer pore of Shaker-IR (Tan et al., 2022). Earlier mutagenesis studies of Shaker-IR suggested that the intra-subunit bond is essential for preventing slow inactivation, while the two inter-subunit bonds are important for structural integrity but not slow inactivation because their disruption only leads to non-functional channels or channels with normal slow inactivation (Pless et al., 2013; Lueck et al., 2016). Disruption of inter-subunit hydrogen bonds in the Kv1.2 channel also result in non-functional channels, highlighting their requirement for structural integrity (Zhang et al., 2021). In mouse Kv1.3, the D402N mutation (D449 in human Kv1.3) significantly accelerates C-type inactivation (Aiyar et al., 1996) possibly because the intra-subunit hydrogen bond (W436-D449 in human Kv1.3) is broken. These studies suggest that rupture of the intra-subunit bond that fastens the SF to the pore helix promotes slow inactivation in Kv channels.

In the wild-type Kv1.3 structure (Apo-Kv1.3), the voltage sensors are in the depolarized position and the inner (activation) gate is open indicating that the channel is activated (Tyagi et al., 2022). In Apo-Kv1.3, the critical intra-subunit bond (W436-D449) tying the SF to the pore helix is broken (Tyagi et al., 2022) (Figure 1F). The unfettered SF flips outwards toward the external solution. The SF residue Y447 swings out by about 11 Å and forms an intra-subunit hydrogen bond with H451 (Tyagi et al., 2022), a residue in the outer vestibule known to play a critical role in Kv1.3’s C-type inactivation (Grissmer and Cahalan, 1989b; a;Busch et al., 1991; Panyi et al., 1995; Nguyen et al., 1996; Jager et al., 1998; Hanson et al., 1999; Somodi et al., 2004). This reorganization results in dilation (4–5 Å) at sites S1 and S2 in the outer SF (Figure 2D) and absence of K^+^ occupancy from site S2 (Figure 1F). All-atom MD simulations of the pore of Apo-Kv1.3 following a voltage pulse (700 mV; which admittedly is large and not physiological) show rapid breaking of key intra-subunit hydrogen bonds (H451–Y447, H451–D449), leading to significant distortions of the extracellular end of the SF, elimination of the S1 and S2 K^+^ binding sites, and flooding of the SF with water during the simulation (Tyagi et al., 2022). This causes rapid stochastic outward permeation of K^+^, during which ions are initially located at the S3 and S4 binding sites and get knocked on by incoming K^+^ ions into the outer SF, where after hydration they leave the pore (Tyagi et al., 2022). This process does not resemble conventional single-file K^+^-selective knock-on conduction (Tyagi et al., 2022). The stochastic ion conduction observed in Apo-Kv1.3 may be caused by ion movements due to structural relaxations of the SF as Kv1.3 adapts to the large applied voltage on the relatively short timescale of the MDS run. The ion configuration of Apo-Kv1.3’s SF (Tyagi et al., 2022) resembles the open-inactivated I_1_ conformation of KcsA (Cuello et al., 2010a, with both having K^+^ at sites S1, S3, and S4 in the SF. However, the dimensions of the SF of Apo-Kv1.3 and I_1_-KcsA, are different. Apo-Kv1.3 is dilated by 4–5 Å at sites S1 and S2 in the outer SF and the inner SF is unchanged (Figure 2D) compared to the open-conducting conformations of KvChim (Figure 2A) and Shaker-IR (Figure 2B). In contrast, I_1_-KcsA is narrowed by 2 Å at site S2 in the outer pore (Figure 2G) compared to the open-conducting conformation of KcsA (Figure 2F) (Cuello et al., 2010b). Interestingly, the dimension of the open inner gate of Apo-Kv1.3 (Cα-Cα at V476 = 15.3 Å) (Tyagi et al., 2022) is similar to the dimension of the inner gate of the open-conducting conformation of KcsA (15 Å) and not the I_1_-KcsA conformation (17 Å) when allosteric coupling is triggered (Cuello et al., 2010b). Allosteric coupling should not therefore be obvious in in the open-inactivated Apo-Kv1.3 structure as evidenced by the similar orientation and dimensions of the key coupling residues in Apo-Kv1.3 (T444-I472; PDB:7WF4) versus the open-conducting conformations of Shaker-IR (T442-I470; PDB:7SJ1) and KvChim (T389-I417; PDB:2R9R). Existing data suggest that slow inactivation might involve an ensemble of inactivated states (including shallow and deeply inactivated states), and a single C-type inactivated conformation does not exist (Yang et al., 1997; Loots and Isacoff, 1998). Apo-Kv1.3 and I_1_-KcsA may therefore represent two different shallow open-inactivated conformations. Alternatively, voltage-dependent slow inactivation in Apo-Kv1.3 may be different from pH-dependent C-type inactivation in KcsA. It is conceivable that Kv1.3 can enter deep open-inactivated states, but this would require the inner gate to open to the width (23–32 Å) seen in I_2_-KcsA (Cuello et al., 2010b).

For Shaker-IR, a C-type inactivated conformation of the SF was achieved with the W434F mutation, which eliminates ionic current by causing Shaker-IR channels to be permanently inactivated (Perozo et al., 1993; Yang et al., 1997). Inactivation in the W434F mutant differs from physiological C-type inactivation in Shaker-IR channels. Strong hyperpolarization removes physiologically relevant C-type inactivation in Shaker-IR (Panyi and Deutsch, 2006), but it does not remove inactivation in hetero-tetramers containing two W434F mutant subunits (Yang et al., 1997). Further, slow inactivated Shaker-IR is blocked by the scorpion peptide agitoxin with equal potency as the channel’s closed state (Liu et al., 1996), whereas the W434F mutant is significantly less sensitive to block by agitoxin (Aggarwal, 1996; Yang et al., 1997). These differences suggest that the W434F mutant is in a deep inactivated state. In the Shaker-IR W434F structure, the voltage sensors are in the depolarized position and the inner (activation) gate is open, indicating that the channel is fully activated (Tan et al., 2022). Since the W434F mutant is non-conductive whether the inner gate is open or closed, it likely bypasses the mechanisms that couple opening of the inner (activation) gate to the conformational changes in the SF related to slow inactivation. The W434F mutation disrupts the intra-subunit hydrogen bond (W434-D447) that tethers the SF to the pore helix (Tan et al., 2022; Tyagi et al., 2022) (Figure 1E), and allows the freed SF to move outwards by about 5 Å (Tan et al., 2022; Tyagi et al., 2022) (Figure 1E). SF residues Y445 and D447 reorient away from the interior of the pore with D447 positioned near T449 (Figure 1E) (Tan et al., 2022; Tyagi et al., 2022), a residue in the outer vestibule critical for Shaker’s C-type inactivation (Choi et al., 1991; Lopez-Barneo et al., 1993; Yellen et al., 1994; Liu et al., 1996). An additional intra-subunit bond (F434-M448) stabilizes the open-inactivated conformation of Shaker-IR W434F (Figure 1E). This reorganization results in dilation (4–5 Å) at sites S1 and S2 in the outer SF, and the loss of coordination of K^+^ at sites S1 and S2, with K^+^ occupancy only at sites S3 and S4 (Figure 1E, Figure 2C). All-atom MD simulations following a voltage pulse (300 mV) resulted in no permeation events during a 2.5-μs-long run, although voltage jumps to higher voltages caused K^+^ permeation events (Tan et al., 2022). Other MD simulation studies on Shaker-IR W434F with the inner (activation) gate set at “fully” opened (23–32 Å) show a pinching (constriction) at the outermost S0 and S1 binding sites (Li et al., 2021b). This constriction contrasts with the dilation seen in the outer SF of the Shaker-IR W434F structure with the inner gate opened to 15 Å (Tan et al., 2022). Importantly, the conformation of Shaker-IR W434F’s SF (Tan et al., 2022) resembles that of Apo-Kv1.3 (Tyagi et al., 2022), suggesting that both are in the open-inactivated conformation. However, the structural changes seen at the extracellular side of Shaker-IR W434F are large (Figure 2C) and not compatible with the constricted outer vestibule seen in Shaker-IR that has undergone physiological slow inactivation (Yellen et al., 1994; Liu et al., 1996). The projection of D447 into Shaker-IR W434F’s outer vestibule (Figure 2C) will likely sterically hinder peptide toxin binding and may explain the reduced agitoxin-sensitivity of the mutant (Aggarwal, 1996; Yang et al., 1997). In contrast, the changes in the outer vestibule of Apo-Kv1.3 are more subtle (Figure 2D). The ∼2–3 Å narrowing of the outer vestibule at site S0 of Apo-Kv1.3 (Figure 2D), compared to KvChim (Figure 2A) and Shaker-IR (Figure 2B), is consistent with the narrowed outer vestibule in C-type inactivated Shaker-IR (Yellen et al., 1994; Liu et al., 1996). An additional difference is that Apo-Kv1.3 contains three K^+^ in the SF while Shaker-IR W434F contains two K^+^ (Figures 2C,D). Two K^+^ also occupy the SF in the I_2_ open-inactivated conformation of KcsA, but these K^+^ are at sites S1 and S4 (Figure 2H) (Cuello et al., 2010b) instead of sites S3 and S4 seen in Shaker-IR W434F (Figure 2C) (Tan et al., 2022). Further, Shaker-IR W434F’s outer SF (at sites S1 and S2) is dilated and its inner SF (at sites S3 and S4) remains unchanged like Apo-Kv1.3 (Figure 2C) (Tan et al., 2022), whereas I_2_-KcsA has a dilated inner SF (Figure 2H) (Cuello et al., 2010b). In summary, Shaker-IR W434F may represent a deep open-inactivated state which is different from the deep open-inactivated I_2_-KcsA conformation, and also from the shallow open-inactivated conformations of Apo-Kv1.3 and I_1_-KcsA.

## Dalazatide-Induced Changes in the Filter May Resemble Recovery From C-Type Inactivation

Dalazatide (ShK-186), an analog of the sea anemone toxin ShK, blocks Kv1.3 with picomolar affinity and specificity. It has advanced to human trials for the treatment of autoimmune and neuroinflammatory disorders (Tarcha et al., 2012; Tarcha et al., 2017; Wulff et al., 2019). In the high-resolution structure of Kv1.3-Kvβ2 bound to dalazatide (Dal-Kv1.3) a molecule of dalazatide can be seen bound to the extracellular side of Kv1.3’s SF, but a model of dalazatide could not be built into its density due to the symmetry mismatch arising from a non-symmetrical dalazatide molecule binding to a fourfold symmetrical Kv1.3 (Tyagi et al., 2022). In the Dal-Kv1.3 structure, the voltage sensors are in the depolarized position and the inner (activation) gate is open, indicating that the channel is activated (Tyagi et al., 2022). The Dal-Kv1.3 structure reveals dramatic reorganization of the outer pore compared to Apo-Kv1.3 (Tyagi et al., 2022). This reorganization appears to partially reverse the structural arrangements responsible for slow inactivation. Dalazatide binding to the channel’s outer vestibule reconfigures the dilated outer SF of Apo-Kv1.3 into a narrower architecture observed in the open-conformations of KvChim and Shaker-IR (Figures 2A,B,D,E). Large movements of residues in Kv1.3’s outer pore break intra-subunit hydrogen bonds (Y447-H451, D449-H451) that tie the SF in the outward position in Apo-Kv1.3 (Figure 1F). The freed SF swings inwards and attaches to the pore helix by two inter-subunit hydrogen bonds (W437-Y447; T441-Y447) (Figure 1G) equivalent to those in KvChim and Shaker-IR (Figures 1C,D). However, the critical intra-subunit W436-D449 bond between the pore helix and SF is missing in Dal-Kv1.3, suggesting an incomplete reversal from the slow inactivated conformation. Instead, a third inter-subunit bond (H451–D449) contributes to stability of the narrowed outer pore of Dal-Kv1.3 (Figure 1G). The reordered filter of Dal-Kv1.3 is narrower than the dilated SF in Apo-Kv1.3 by ∼4–5 Å (Figures 2D,E). Ion binding sites S2-S4 are occupied in Dal-Kv1.3 (Figure 2E). A density observed over site S1 is likely to be a pore-occluding dalazatide residue (Tyagi et al., 2022) similar to the pore-occluding toxin residue seen in the charybdotoxin-bound structure of KvChim (Banerjee et al., 2013). MD simulation studies of Dal-Kv1.3 (with dalazatide removed) demonstrate single-file knock-on conduction of K^+^ through the pore in contrast to stochastic conduction of K^+^ in Apo-Kv1.3 (Tyagi et al., 2022). These dalazatide-induced changes in Kv1.3, especially the movement of Y447, are similar to the conformational changes induced by scorpion peptide kaliotoxin in a Kv1.3–KcsA chimera grafted with Kv1.3’s P-loop and detected with solid state NMR (Lange et al., 2006; Zachariae et al., 2008). Perhaps, pore-blocking peptides induce constriction of the outer pore to optimize their fit into Kv1.3, or they may preferentially bind to the resting state of the channel and prevent inactivation to the extent seen in Apo-Kv1.3. In support, C-type inactivation reduces the affinity of dalazatide and kaliotoxin for Kv1.3 possibly because these peptides cannot fit snugly into the dilated outer pore of the inactivated channel (Zachariae et al., 2008; Tyagi et al., 2022). The kaliotoxin-induced conformational changes in KcsA-Kv1.3 are suggested to be structurally and functionally related to recovery from C-type inactivation (Zachariae et al., 2008). Future studies could explore whether recovery from C-type inactivation involves inward movement and narrowing of the outer SF.

## Summary

Kv channels transition from closed to open-conducting to non-conducting inactivated conformations (Figure 2I). In the closed state, the inner gate is closed. Upon channel activation, the opening of the inner gate allows the efflux of K^+^ through the channel pore. C-type inactivation of both human Kv1.3 and fly Shaker-IR W434F begins with the rupture of the intra-subunit hydrogen bond that tethers the SF to the pore helix. The untethered SF swings outwards, causing the outer pore to dilate and perturb ion permeation. Since Kv channels are likely to exist in many slow inactivated states, the structures discussed are representative of states specific for Kv1.3 and Shaker-IR. Recovery from inactivation may entail a reversal of this process. Such a reversal occurs, at least in part, after the binding of dalazatide to Kv1.3 (Figure 1G, Figure 2E). The SF flips inward, the pore narrows, and the two inter-subunit hydrogen bonds that stabilize the outer pore of Dal-Kv1.3 are also found in open-conducting conformations of KvChim and Shaker-IR (Figures 1C,D,G). However, the critical intra-subunit W436-D449 bond that is required to tether the SF to the pore helix is absent (Figure 1G), suggesting that recovery is incomplete.

In future studies, it will be instructive to examine how the conformation of the SF is impacted by changes that slow or accelerate C-type inactivation (Grissmer and Cahalan, 1989a; b;Nguyen et al., 1996), and whether the non-conducting state that develops following the removal of external K^+^ (Pardo et al., 1992; Lopez-Barneo et al., 1993; Jager et al., 1998) resembles the C-type inactivated conformation. It will also be important to determine why C-type inactivation is affected by changes below the SF, for example by drugs that bind in the central cavity (Psora-4), or by mutations in the S6 helix (e.g. Kv1.3 A413V = A465V in full-length human Kv1.3; Shaker-IR V478W; and KvChim V406W) (Panyi et al., 1995; Vennekamp et al., 2004; Zimin et al., 2010; Pau et al., 2017). A high-resolution crystal structure of KvChim V406W revealed an essentially unchanged SF with one of the two structures showing a slight narrowing of the SF at the S1 K^+^ binding site (Pau et al., 2017). Perhaps a different mechanism underlies C-type inactivation caused by changes in the outer pore versus below the SF.
